# *Citrus* Pomace as a Source of Plant Complexes to Be Used in the Nutraceutical Field of Intestinal Inflammation

**DOI:** 10.3390/antiox13070869

**Published:** 2024-07-19

**Authors:** Mariarosaria Ingegneri, Maria Rita Braghini, Michela Piccione, Cristiano De Stefanis, Manuela Mandrone, Ilaria Chiocchio, Ferruccio Poli, Martina Imbesi, Anna Alisi, Antonella Smeriglio, Domenico Trombetta

**Affiliations:** 1 Department of Chemical, Biological, Pharmaceutical and Environmental Sciences, University of Messina, Viale Ferdinando Stagno d’Alcontres 31, 98166 Messina, Italy; mariarosaria.ingegneri@unime.it (M.I.); martina.imbesi@studenti.unime.it (M.I.); domenico.trombetta@unime.it (D.T.); 2 Research Unit of Genetics of Complex Phenotypes, Bambino Gesù Children’s Hospital, Istituti di Ricovero e Cura a Carattere Scientifico (IRCCS), 00165 Rome, Italy; mariarita.braghini@opbg.net; 3 Core Facilities, Bambino Gesù Children’s Hospital, Istituti di Ricovero e Cura a Carattere Scientifico (IRCCS), 00165 Rome, Italy; michela.piccione@opbg.net (M.P.); cristiano.destefanis@opbg.net (C.D.S.); 4 Department of Pharmacy and Biotechnology (FaBit), Alma Mater Studiorum, University of Bologna, Via Irnerio 42, 40126 Bologna, Italy; manuela.mandrone2@unibo.it (M.M.); ilaria.chiocchio2@unibo.it (I.C.); ferruccio.poli@unibo.it (F.P.)

**Keywords:** *Citrus* by-products, food-grade extracts, phytochemistry, primary metabolites, secondary metabolites, in vitro simulated gastro-duodenal digestion, intestinal bioaccessibility, antioxidant activity, anti-inflammatory activity, nutraceutics

## Abstract

This study aims to recover the main by-product of *Citrus* fruits processing, the raw pomace, known also as *pastazzo*, to produce plant complexes to be used in the treatment of inflammatory bowel disease (IBD). Food-grade extracts from orange (OE) and lemon (LE) pomace were obtained by ultrasound-assisted maceration. After a preliminary phytochemical and biological screening by in vitro assays, primary and secondary metabolites were characterized by proton nuclear magnetic resonance (^1^H-NMR) and liquid chromatography coupled to diode array detection and electrospray ionization mass spectrometry (LC-DAD-ESI-MS) analyses. The intestinal bioaccessibility and antioxidant and anti-inflammatory properties were investigated by in vitro simulated gastro-intestinal digestion followed by treatments on a lipopolysaccharide (LPS)-stimulated human colorectal adenocarcinoma cell line (Caco-2). The tight junctions-associated structural proteins (ZO-1, Claudin-1, and Occludin), transepithelial electrical resistance (TEER), reactive oxygen species (ROS)-levels, expression of some key antioxidant (*CAT*, *NRF2* and *SOD2*) and inflammatory (*IL-1β*, *IL-6*, *TNF-α*, *IL-8*) genes, and pNFkB p65 nuclear translocation, were evaluated. The OE and LE digesta, which did not show any significant difference in terms of phytochemical profile, showed significant effects in protecting against the LPS-induced intestinal barrier damage, oxidative stress and inflammatory response. In conclusion, both OE and LE emerged as potential candidates for further preclinical studies on in vivo IBD models.

## 1. Introduction

Over the past decades, it has been widely demonstrated that the consumption of functional foods such as fresh fruit and vegetables and their processed products is essential to ensure our body’s vitality and health. However, the increased consumption, especially of processed products such as juices, extracts, centrifuges, fourth range products, etc., implies a significant increase in process wastes, which are often very expensive to dispose [1,2].

Despite this, these waste products can still be considered precious raw materials for producing plant complexes or isolating pure molecules to be recovered and used both in the agri-food chain as well as in the nutraceutical field.

*Citrus* represent some of the most important and valued fruit crops in the world [3]. Consumed by humans since ancient times, these fruits are well-known for their health effects thanks to the wide range of hydrophilic and lipophilic bioactive compounds they contain [4]. World production of *Citrus* fruits has grown steadily over the past three decades [5]. Data provided by the Food and Agriculture Organization Corporate Statistical Database (FAOSTAT) reveal that about 150 million tons of *Citrus* fruits are produced globally every year [1]. Italy, after Spain and Egypt, holds the third place in the ranking of the main *Citrus* fruit-producing countries in Europe [3]. Among the Italian regions, Sicily holds the record in *Citrus* fruits production, comprising more than 60% of the entire national market [6,7]. Oranges and lemons are the most cultivated and marketed *Citrus* fruits and consequently also the most processed, producing annually about 230.000 tons of raw pomace (also known as *pastazzo*)*,* a by-product consisting of flavedo, albedo, seeds and pulp fruit residues [3,8].

Despite being a waste product, *Citrus* raw pomace is a rich source of value-added compounds such as polyphenols, polysaccharides, organic acids, terpenes, amino acids, minerals, vitamins and carotenoids [8]. In this context, over the last decades, research has focused on the study of alternative applications which would allow its recovery from a circular economy perspective such as its use as a fertilizer, in animal feed or to produce biofuels [3,4,9]. However, the use of this type of waste currently lacks evidence for application in the health field, although many bioactive compounds typical of *Citrus* fruit, in particular flavanones, have proven to be very promising for their strong antioxidant and anti-inflammatory properties [3,10], particularly when used in combination for their proved synergistic activity [11]. As suggested by experimental studies, the properties of these natural compounds could have valuable preventive and therapeutic effects on several noncommunicable diseases (NCDs), such as metabolic dysfunction associated steatotic liver disease and type 2 diabetes [12,13].

Among the NCDs that provide major contributions to the reduction of the quality of life and life expectancy, there are inflammatory bowel diseases (IBD), a group of pathologies including Crohn’s disease (CD) and ulcerative colitis (UC). IBD affects mainly adolescents and middle-aged people, and in 2017 a Global Burden of Disease study estimated approximately 6.8 million cases of IBD globally [14]. IBDs are characterized by recurrent non-infectious gastro-intestinal tract inflammation [15], whose symptoms may include abdominal pain, diarrhea, weight loss and rectal bleeding [16,17]. Although the etiology remains unknown, it is possible to speculate that, in genetically predisposed individuals, the onset of IBD may be due to a disruption of the host immune response and intestinal commensal bacteria balance. Furthermore, environmental, behavioral and dietary factors play a key role in the onset of IBD, so much so that they are referred to as multifactorial diseases [18,19]. Current pharmacological approach consists in symptomatic treatments and complication-managing drugs such as antibiotics, corticosteroids, immunosuppressants and tumor necrosis factor α (TNF-α)-inhibitors, which however often fail to achieve and sustain remission, and can even cause serious side effects [16,17]. Given their chronic and progressive nature and the healthcare costs, the rapidly increasing incidence of IBD has become a major socio-economic concern [20]; the search for alternative therapies represents a challenge for research studies on IBD [21]. Indeed, in recent years, there has been a significant increase in studies on IBD and natural substances, to find alternatives to conventional therapy, but to date, research has mainly focused on natural products and extracts obtained from edible parts, and plant complexes obtained from the agri-food waste have rarely been considered.

In this context, the aim of the present study was to investigate the phytochemical profile and intestinal bioaccessibility of standardized and titrated food-grade extracts of conventional blond orange and organic lemon raw pomace, and to test their antioxidant and anti-inflammatory properties by in vitro cell-free and cell-based assays to select plant complexes potentially useful for nutraceutical purposes in the context of IBD.

## 2. Materials and Methods

### 2.1. Sample Preparation

Raw pomace samples of conventional blond orange (*Citrus sinensis* (L.) Osbeck cultivar “Valencia”) coming from Carlentini, Lentini and Messina, and organic lemon (*Citrus limon* L. Burm. cultivar “Femminello”) coming from Syracuse, were kindly provided by Simone Gatto S.r.l., a leader Sicilian company in the production of *Citrus* essential oils and juices of absolute purity, which currently distributes its processing products in 27 countries. This company was also chosen for the quality of the starting material, which is guaranteed by the selection of *Citrus* groves based on sustainable supply chain, fair price, low environmental impact, compliance to the varieties and pesticide control.

To standardize the extraction process, three different batches for each *Citrus* raw pomace type were supplied and processed independently. Samples were cryo-powdered in liquid nitrogen with a blade analytical mill (A11, IKA^®^-Werke GmbH & Co. KG, Staufen, Germany) to inhibit enzymatic activity, thus preserving the native phytochemical profile. Food-grade hydroalcoholic extracts were obtained by ultrasound-assisted extraction (matrix/solvent 1:10, *w*/*v*) at room temperature (RT) according to Smeriglio et al. [22] using four different ethanol/water ratios: 50:50, 60:40, 70:30 and 80:20 *v*/*v*, respectively. The extraction procedure was repeated three times, and the obtained supernatants were collected and dried, at RT and in the dark, by rotary evaporator (Büchi R-205, Cornaredo, Italy). Dry orange and lemon raw pomace extracts (OE and LE, respectively) were stored in a vacuum glass desiccator overnight with anhydrous sodium sulfate. After calculating the extraction yield, both extracts were stored in burnished sealed vials with nitrogen headspace. At the time of the analyses, fresh DMSO stock solutions were prepared and then diluted in Milli-Q water to carry out all cell-free and cell-based in vitro assays.

### 2.2. Phytochemical Analyses

#### 2.2.1. Proton Nuclear Magnetic Resonance (^1^H-NMR) Analysis

Deuterium oxide (D_2_O, 99.90% D) and CD_3_OD (99.80% D) were purchased from Eurisotop (Cambridge Isotope Laboratories, Inc., Saint-Aubin, France). The standard 3-(trimethylsilyl)-pro-pionic-2,2,3,3-*d*_4_ acid sodium salt (TMSP), sodium phosphate dibasic anhydrous, sodium phosphate monobasic anhydrous as well as all other chemicals and solvents were purchased from Sigma-Aldrich Co. (St. Louis, MO, USA).

^1^H NMR spectra were recorded at 25 °C on a Varian Inova instrument (equipped with a reverse triple-resonance probe) operating at a frequency of 600.13 MHz, and using MeOH-d4 as internal lock. Each ^1^H NMR spectrum consisted of 256 scans (corresponding to 16 min) with a relaxation delay (RD) of 2 s, acquisition time 0.707 s and spectral width of 9595.8 Hz (corresponding to δ 16.0). A presaturation sequence (PRESAT) was used to suppress the residual water signal at δ 4.83 (power = −6 dB, presaturation delay 2 s).

OE and LE (700 μL, 10 mg/mL) solubilized in phosphate buffer (90 mM; pH 6.0) in D_2_O (containing 0.1% TMSP) and CD_3_OD (1:1, *v*/*v*) were transferred into NMR tubes.

Five different extracts were measured to test reproducibility. Semi-quantitative analysis was performed by integration of the diagnostic signals of the compounds of interest in comparison with TMSP internal standard. Compounds identification was based on the literature and in-house database [23,24].

#### 2.2.2. Secondary Metabolites Screening by Colorimetric Assays

##### Total Phenolic Compounds (TPC)

Total phenolics were quantified according to Ingegneri et al. [25]. Briefly, 10 μL of OE and LE (0.625–5.0 mg/mL) were added to 90 µL of Milli-Q water and mixed 1:1 (*v*/*v*) with Folin–Ciocalteu reagent. After 3 min, 100 μL 10% sodium carbonate were added and samples incubated in the dark at RT for 60 min, shaking every 10 min. Absorbance was read at 785 nm by using a Multiskan™ GO Microplate Spectrophotometer (Thermo Scientific, Waltham, MA, USA) against Milli-Q water as blank. Gallic acid was used as a reference compound (0.075–0.6 mg/mL), and results were expressed as g of gallic acid equivalents (GAE)/100 g dry extract (DE). 

##### Total Flavonoid Compounds (TFC)

Total flavonoids were quantified according to Lenucci et al. [26]. Briefly, 50 μL of OE and LE (1.25–10 mg/mL) were added to 450 μL of Milli-Q water and 30 μL of 5% NaNO_2_. After 5 min, 60 μL of 10% AlCl_3_ were added, and samples incubated for 6 min at RT. Two hundred microliters of 1 M NaOH and 210 μL of Milli-Q water were added, and samples were vortex-mixed. The absorbance was recorded at 510 nm by an UV-1601 spectrophotometer (Shimadzu, Kyoto, Japan). Rutin was used as a reference standard (0.125–1.0 mg/mL), and results were expressed as g of rutin equivalents (RE)/100 g DE.

##### Vanillin Index

Vanillin index is a specific assay useful to detect flavan-3-ols and dihydrochalcones that have a single bond at the 2,3-position, and free meta-oriented hydroxy groups on the B ring. Briefly, 0.5 mL of OE and LE (20 mg/mL) were added to 1.5 mL 0.5 M sulfuric acid and loaded onto a conditioned Sep-Pak C18 cartridge (Waters, Milan, Italy), which was then washed with 2.0 mL of 5.0 mM sulfuric acid. Samples were eluted with 5.0 mL of methanol, and 1 mL of each eluate was added to 6.0 mL of 4% vanillin methanol solution and incubated at 20 °C for 10 min. HCl (3 mL) was added, and after 15 min at RT, the absorbance was recorded at 500 nm [27] using the same instrument and blank reported in Total Flavonoid Compounds (TFC) Section. Catechin was used as a reference standard (0.0625–0.50 mg/mL). Results were expressed as g of catechin equivalents (CE)/100 g DE.

##### Proanthocyanidins

Proanthocyanidins were quantified by hot acid hydrolysis [27], diluting OE and LE (40 mg/mL) in 0.05 M sulfuric acid (2 mL). Solutions were loaded onto conditioned Sep-Pak C18 cartridges (Waters, Milan, Italy). Proanthocyanidin-rich fractions obtained were eluted with methanol (3 mL) and collected in 100 mL round bottom flasks shielded from light and containing 9.5 mL of absolute ethanol. After this, 12.5 mL of 300 mg/L FeSO_4_ · 7H_2_O hydrochloric acid solution was added and samples left to reflux for 50 min. After cooling, the absorbance was recorded at 550 nm using the same instrument and blank reported in Total Flavonoid Compounds (TFC) Section. To subtract the starting anthocyanins content of samples, the absorbance of samples prepared under the same conditions, but cooled in ice instead of warmed, was subtracted from that of the heated samples to obtain the net value of absorbance. Proanthocyanidins concentration was expressed as g of cyanidin chloride equivalents (ε = 34,700) (CyE)/100 g DE.

#### 2.2.3. LC-DAD-ESI-MS Analysis

OE and LE secondary metabolites were characterized by a previously validated LC-DAD-ESI-MS method [10,27]. Separation was carried out at 25 °C using Luna Omega PS C18 column 150 mm × 2.1 mm, 5 µm (Phenomenex, Torrance, CA, USA). The following elution program, using 0.1% formic acid (Solvent A) and acetonitrile (Solvent B) as mobile phase, was used: 0–3 min, 0% B; 3–9 min, 3% B; 9–24 min, 12% B; 24–30 min, 20% B; 30–33 min, 20% B; 33–43 min, 30% B; 43–63 min, 50% B; 63–66 min, 50% B; 66–76 min, 60% B; 76–81 min, 60% B; 81–86 min, 0% B and equilibrated 4 min. Five microliters of OE and LE were injected, recording the UV–Vis spectra from 190 to 600 nm. Acquisition was carried out at different wavelengths (260, 280, 292, 330, 370 and 520 nm) to identify all polyphenols classes. For the Agilent 6320 ion trap (Agilent Technologies, Santa Clara, CA, USA), both negative and positive electrospray ionization (ESI) mode was selected setting the capillary voltage, nebulizer (N_2_) pressure, drying gas temperature, drying gas flow and skimmer voltage as follows: 3.5 kV, 40 psi, 350 °C, 9 L/min and 40 V. Acquisition was carried out in full-scan mode (90–2000 m/z). Data were acquired by Agilent ChemStation software version B.01.03 and Agilent trap control software version 6.2 (Agilent Technologies, Santa Clara, CA, USA).

Identification was carried out by comparing the retention times, UV–Vis and MS spectra of each analyte with those of commercially available standards, literature data and UV–Vis and open-source mass spectra databases. Chromatograms acquired at 330 nm were used to quantify, by using external calibration curves of the HPLC-grade reference standards (purity ≥ 98%, Extrasynthase, Genay, France), the chosen phytochemical markers hesperidin and narirutin for OE, eriocitrin and hesperidin for LE.

### 2.3. In Vitro Simulated Gastrointestinal Digestion

The in vitro simulated gastrointestinal digestion of OE and LE was carried out according to the INFOGEST protocol [28].

OE and LE solution were added (1:1, *v*/*v*) to a simulated gastric fluid (SGF) consisting of 1.25X electrolytes stock solution, 0.3 M calcium dichloride dihydrate, porcine pepsin (2000 U/mL), gastric lipase (60 U/mL), Milli-Q water and 5 M HCl for pH adjustment. Samples were then incubated under agitation at pH 3.0 for 2 h. The gastric chyme was then diluted (1:1, *v*/*v*) with simulated intestinal fluid (SIF), consisting of 1.25X electrolytes stock solution, 0.3 M calcium dichloride dihydrate, porcine trypsin (100 U/mL), bovine chymotrypsin (25 U/mL), porcine pancreatic α-amylase (200 U/mL), porcine pancreatic lipase (2000 U/mL), porcine pancreatic colipase (4000 U/mL), 10 mM bile salts, Milli-Q water and 5 M NaOH for pH adjustment. Samples were then incubated under agitation at pH 7 for a further 2 h. At the end of the procedure, according to the INFOGEST protocol for bioaccessibility of phytochemicals [28], OE and LE digesta were centrifuged and filtrated using a 0.20 μm nylon syringe filter, and immediately stored at −80 °C until subsequent analyses. Extraction of digesta samples for phytochemical analyses were carried out according to Denaro et al. [10].

### 2.4. In Vitro Antioxidant and Anti-Inflammatory Assays

The antioxidant and anti-inflammatory activity of OE and LE were evaluated by several in vitro spectrophotometric and spectrofluorimetric assays based on different mechanisms and reaction environments. Results were expressed as inhibition (%) of the oxidative/inflammatory activity by calculating the half-inhibitory concentration (IC_50_) and the respective confidence limits (C.L.) at 95% by Litchfield and Wilcoxon’s test (PHARM/PCS 4, MCS Consulting, Wynnewood, PA, USA). The following reported concentration ranges refer to final concentrations in the reaction mixture.

#### 2.4.1. 2,2-Diphenyl-1-Picrylhydrazyl (DPPH) Assay

The reaction mixture, consisting of OE (0.25–2.0 mg/mL) or LE (0.125–1.0 mg/mL) and fresh 2.50 mg/mL DPPH methanol solution (1:40, *v*/*v*) was mixed and incubated in the dark at RT for 20 min [25]. The absorbance was recorded at 517 nm using the same instrument and blank reported in Total Phenolic Compounds (TPC) Section. Trolox was used as a reference standard (2.5–20.0 μg/mL).

#### 2.4.2. Trolox Equivalent Antioxidant Capacity (TEAC) Assay

The blue-green cationic radical solution, obtained by incubating at RT for 12 h the 1.7 mM diammonium salt of 2,20-azino-bis (3-ethylbenzothiazolin-6-sulphonic acid (ABTS)) with 4.3 mM K_2_S_2_O_8_, was diluted with Milli-Q water to obtain an absorbance of 0.7 ± 0.02 at 734 nm, and used within 4 h. Ten microliters of OE and LE (31.25–250.0 µg/mL) were added to the radical solution (200 μL) and incubated at RT for 6 min [25]. The absorbance decrease was recorded at 734 nm using the same instrument and blank reported in Total Phenolic Compounds (TPC) Section. Trolox was used as a reference standard (1.25–10.0 µg/mL).

#### 2.4.3. Ferric-Reducing Antioxidant Power (FRAP) Assay

OE and LE (62.5–500.0 µg/mL) were added to fresh pre-warmed (37 °C) working reagent (1:20, *v*/*v*), consisting of 300 mM buffer acetate (pH 3.6), 10 mM 2,4,6-Tris(2-pyridyl)-s-triazine (TPTZ) dissolved in 40 mM HCl and 20 mM iron(III) chloride, and incubated for 4 min at RT in the dark [25]. The absorbance was recorded at 593 nm using the same instrument and blank reported in Total Phenolic Compounds (TPC) Section. Trolox was used as a reference compound (1.25–10.0 μg/mL).

#### 2.4.4. ORAC

OE and LE (1.25–10.0 µg/mL) were added to fresh 117 nM fluorescein phosphate buffer saline (PBS) solution and incubated for 15 min at 37 °C. After this, 40 mM 2,2′-azobis(2-methylpropionamidine) dihydrochloride (AAPH) PBS solution was added, achieving the following reagents ratio (1:6:3 *v*/*v*/*v*, respectively) [25]. The fluorescein decay was recorded every 30 s for 90 min (λ_ex_ 485; λ_em_ 520) by a microplate reader (FLUOstar Omega, BMG LABTECH, Ortenberg, Germany). Trolox was used as a reference compound (0.25–2.0 μg/mL).

#### 2.4.5. β-Carotene Bleaching (BCB) Assay

The BCB assay was carried out according to Smeriglio et al. [2] with some modifications [2]. Briefly, 80 μL of OE and LE (62.5–500.0 μg/mL) were added to 2 mL of a β-carotene emulsion consisting of β-carotene chloroform solution (2.5 mg/mL), 4 µL of linoleic acid, and 100 μL of Tween-40. A β-carotene free emulsion was used as a negative control, whereas a β-carotene emulsion with Milli-Q water was used as a blank. Samples were incubated for 120 min at 50 °C in a shaking water bath, monitoring the absorbance decay every 20 min at 470 nm, using the same instrument reported in Total Phenolic Compounds (TPC) Section. Butylhydroxytoluene (BHT) was used as reference standard (0.06–0.5 µg/mL).

#### 2.4.6. Iron-Chelating Activity (ICA) Assay

The iron-chelating activity was evaluated according to Smeriglio et al. [2] with some modifications. Briefly, 25 μL of 2.0 mM iron (II) chloride tetrahydrate were added to 50 μL of OE and LE (75.0–600.0 μg/mL, respectively) and incubated at RT for 5 min. Then, 50 μL of 5 mM ferrozine were added and the reaction mixture were diluted to 1.5 mL with Milli-Q water, vortex-mixed, and incubated for 10 min at RT. The absorbance was read at 562 nm using the same instrument and blank reported in Total Flavonoid Compounds (TFC) Section. EDTA was used as a reference standard (1.5–12.0 μg/mL).

#### 2.4.7. Heat-Induced Bovine Serum Albumin Denaturation (ADA)

OE and LE (0.25–2.0 mg/mL and 0.125–1.0 mg/mL, respectively) were added to 0.4% fatty-acid-free bovine serum albumin (BSA) solution and PBS pH 5.3 (4:5:1 *v*/*v*/*v*, respectively) [10]. Once the starting absorbance had been recorded at 595 nm, samples were incubated for 30 min at 70 °C in a shaking water bath, recording the final absorbance at the same wavelength and using the same instrument and blank reported in Total Phenolic Compounds (TPC) Section. Diclofenac sodium was used as a reference standard (3.0–24.0 µg/mL).

#### 2.4.8. Protease-Inhibitory Activity (PIA)

Twenty microliters of OE and LE (31.25–250.0 µg/mL) were added to 12 µL of trypsin (10 µg/mL), 188 µL of Tris-HCl buffer pH 7.5 (25 mM) and 400 µL of casein (0.8%) and incubated for 20 min at 37 °C in a shaking water bath [10]. Perchloric acid (400 µL) was added to stop the reaction. After centrifugation (3500× *g* for 10 min), the absorbance of the supernatants was recorded at 280 nm using the same instrument and blank reported in Total Flavonoid Compounds (TFC) Section. Diclofenac sodium was used as a reference standard (2.0–16.0 µg/mL).

### 2.5. Antioxidant and Anti-Inflammatory Cell-Based Assays

#### 2.5.1. Cell Culture and Treatments

The human colorectal adenocarcinoma Caco-2 cell line purchased from and certified by American Type Culture Collection (ATCC, Manassas, VA, USA) was cultured in Eagle’s Minimum Essential Medium (EMEM) (ATCC) supplemented with 10% fetal bovine serum (Gibco-Thermo Fisher Scientific, Waltham, MA, USA) and 1% penicillin/streptomycin (Euroclone, Milan, Italy) and incubated at 37 °C with 5% CO_2_ in a humidified atmosphere. The medium was changed three times a week and possible mycoplasma contamination was checked by using Venor GeM Advance Mycoplasma Detection KIT (Minerva Biolabs, Berlin, Germany), thus performing all the experiments only in mycoplasma-free cells. For the next experiments, all treatments were added in the culture medium as detailed in the specific methods sections.

#### 2.5.2. Cell Viability

Caco-2 cells were seeded in a 96-multiwell plate at a confluence of 8000 cells per well in quintuplicate and then treated with different concentrations of OE and LE (25, 50, 100, 200, and 250 µg/mL), or lipopolysaccharide (LPS) purified from the Gram-negative *E. coli* 0111:B4 purchased by InvivoGen Europe (Toulouse, France) at different concentrations (1, 10, and 25 µg/mL), alone or in combination. The cell viability was then assessed at two different timepoints (24 and 48 h) by using the cell proliferation kit II-XTT (Roche, Basel, Switzerland) according to the manufacturer’s protocol. Briefly, the kit evaluated the viability of the treated cells by measuring the absorbance at 492 and 620 nm of the water-soluble formazan using the Tecan spectrophotometer (Tecan, Maennedorf, Switzerland).

#### 2.5.3. Cell Proliferation by IncuCyte

Cell proliferation was real-time monitored after treatments with 25, 50, 100, 200, 250 µg/mL OE and LE, or 1, 10, 25 µg/mL LPS, alone or in combination. Approximately 8000 cells per well were seeded in quintuplicate in 96-multiwell plates. Cell proliferation rate assessed by confluency percentage was evaluated using an IncuCyte live-cell analysis system (Sartorius, Göttingen, Germany), acquiring four images per well every 2 h by using a 10× objective lens over a time course of 48 h. Then, the IncuCyte basic software version 2021A (Sartorius, Gottinga, Germany) was used to perform classic confluence analysis.

#### 2.5.4. Transepithelial Electric Resistance (TEER) Measurement

Caco-2 cells were seeded on PET membrane inserts with 0.4 μm pores (Greiner Bio One, Kremsmünster, Austria) placed in a 24-multiwell plate at a density of 3 × 10^5^ cells/cm^2^ and maintained in complete medium until complete differentiation, changing medium three times a week. TEER was then measured to assess the barrier integrity of the monolayer before and after the treatments using the volt-ohm meter Millicell ERS-2 (Merck Millipore, Burlington, MA, USA). The data were presented as percentage of initial values of unit area resistance calculated by dividing resistance values by the effective membrane area. Membrane inserts without cells were used as blank.

#### 2.5.5. Immunofluorescence

Caco-2 cells with differentiated monolayers were fixed after treatments with 4% paraformaldehyde in H_2_O for 10 minutes. Cells were then washed twice with PBS, blocked with 3% BSA in PBS at RT for 30 min, and then incubated with the primary antibodies diluted 1:100 in PBS/BSA 1% overnight at 4 °C (see Table S1 for the list of antibodies used). After two washes with PBS, cells were incubated with the secondary antibody Alexa Fluor 488 and/or Alexa Fluor 555 (Table S1) in PBS/BSA 1% for 1 h at RT. Finally, cells were incubated with 1:10,000 Hoechst in PBS for 10 minutes at RT for nuclear staining. Image acquisition was performed by using the original digital images format acquired with an Olympus Fluoview FV3000 Confocal Laser Scanning Microscope (Olympus, Tokyo, Japan). The region of interest (ROI) was drawn to perform quantitative fluorescence imaging analysis (QFIA) and the intensity average of fluorescence was calculated using ImageJ software, version 1.8.0 (National Institutes of Health, Bethesda, MD, USA).

#### 2.5.6. Intracellular Reactive Oxygen Species (ROS) Levels

Intracellular ROS levels of Caco-2 cells were evaluated by using the chloromethyl derivative of H_2_DCFDA (CM-H_2_DCFDA), often used as a general oxidative stress indicator (Invitrogen-Thermo Fisher Scientific). Briefly, 8000 cells per well were seeded into a 96-multiwell black plate and after the treatments were incubated for 30 min at 37 °C with 10 μM CM-H2DCFDA fluorescent probe, and with 1:3000 Hoechst, used to normalize the cell amounts by nuclear staining. The fluorescence intensity was then measured at 495 nm excitation and 530 nm emission by using a BioTek Synergy H1 microplate reader (Agilent, Santa Clara, CA, USA). Unstained cells were used as control. Representative images of stained cells were acquired using a Leica DMi8 microscope (Leica Camera AG, Wetzlar, Germany).

#### 2.5.7. Real-Time Quantitative Polymerase Chain Reaction (qPCR)

Total RNA was extracted from Caco-2 cells using Total RNA Purification Plus Kit (Norgen Biotek, Thorold, ON, Canada) according to the manufacturer’s instructions. cDNA reverse transcription was conducted using the SuperScript VILO cDNA Synthesis kit (Invitrogen-Thermo Fisher Scientific). qPCR amplification, detection and analysis were performed by QuantStudio 7 Pro RT-PCR System (Applied Biosystems-Thermo Fisher Scientific, Waltham, MA, USA) using TaqMan Universal PCR Master Mix, No AmpErase UNG (Applied Biosystems-Thermo Fisher Scientific). The mRNA level expression of target genes was determined by using specific TaqMan commercial probes by Applied Biosystems-Thermo Fisher Scientific: nuclear factor erythroid 2-related factor 2-Nrf2 gene (*NRE2L2*, Hs00975961_g1, accession number: NM_001145412), catalase gene (*CAT*, Hs00156308_m1, accession number: NM_001752), Superoxide dismutase 2 gene (*SOD2*, Hs00167309_m1, accession number: NM_000636), interleukin (IL)-1β gene (Hs01555410_m1, accession number: NM_000576), IL-6 gene (Hs00174131_m1, accession number: NM_000600), IL-8 gene (Hs00174103_m1, accession number: NM_000584) and tumor necrosis factor (TNF)-α gene (Hs00174128_m1, accession number: NM_000594). The mRNA levels were normalized to endogenous control gene encoding for glyceraldehyde-3-phosphate dehydrogenase (*GAPDH*, Hs02786624_g1, accession number: NM_001256799). The gene expression levels were represented as fold changes versus control and calculated by the ΔΔCt method.

### 2.6. Statistical Analyses

Data were expressed as IC_50_ with respective 95% C.L. (see Section 2.3), as mean ± standard deviation (SD) of three independent experiments in triplicate for in vitro cell-free assays, and of three independent experiments in quintuplicate for in vitro cell-based assays. The statistical significance was evaluated using one-way analysis of variance (ANOVA) followed by Tukey’s test for the phytochemical and in vitro cell-free assays, and 2-tailed Student’s *t* test for cell-based assays. Values of *p* < 0.05 were considered statistically significant. Data analysis was performed with GraphPad Prism 9.0 (GraphPad Software, San Diego, CA, USA).

## 3. Results

### 3.1. Standardization and Titration of OE and LE

With the aim of obtaining a constant phytochemical profile with the maximum concentration of bioactive compunds, thus guaranteeing reproducibility of the biological effects observed, a standardized extraction procedure was developed. To this end, three different batches of orange and lemon raw pomace were supplied and independently extracted with four different solvent ratios (see Section 2.1 for details). The extraction yield obtained, total phenols and flavonoids content, as well as the concentration of the two chosen phytochemical markers (namely hesperidin and narirutin for OE, eriocitrin and hesperidin for LE) were used as critical parameters.

The 80:20 *v*/*v* hydroalcoholic mixture proved to be the best one, not only in terms of extraction yield (11.35 ± 0.36% and 7.30 ± 0.08% for OE and LE, respectively), but also in terms of the greatest concentration of total phenolic compounds (2.41 ± 0.16 g/100 g and 2.46 ± 0.14 g/100 g for OE and LE, respectively), total flavonoids (1.36 ± 0.09 g/100 g and 1.53 ± 0.08 g/100 g for OE and LE, respectively) and concentration of the chosen phytochemical markers (hesperidin 2.36 ± 0.05 g/100 g and narirutin 0.37 ± 0.01 g/100 g for OE, hesperidin 1.20 ± 0.03 g/100 g and eriocitrin 1.14 ± 0.02 g/100 g for LE). Finally, using the chosen extraction process, no statistically significant difference between the different batches of orange and lemon raw pomace was observed for all the considered critical parameters.

### 3.2. Phytochemical Characterization

#### 3.2.1. ^1^H-NMR Profiling

In this work, we measured the ^1^H NMR profiling of orange and lemon raw pomace. This technique is apt to provide an overview of the most abundant compounds present within an extract and it is increasingly employed to investigate complex matrices, especially for metabolomic studies [29].

^1^H NMR profiling is a robust analytical technique relying on easily standardized sample preparation procedures, producing raw data suitable to be recycled and reused. In this context, the storage of the raw ^1^H NMR profiles in a data repository makes them easily available to the scientific community, which, for instance, might use them to build databases or data analysis models capable of making predictions based on the ^1^H NMR profile.

**Figure 1:**
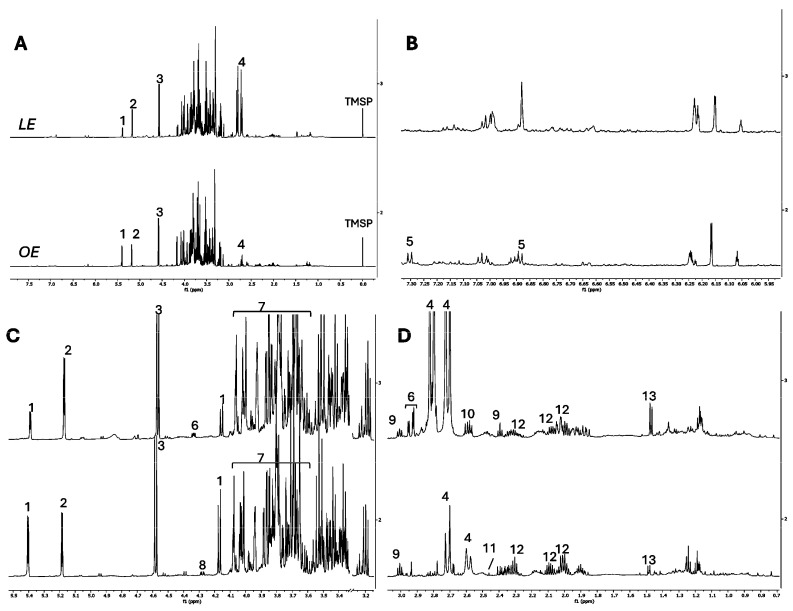
^1^H NMR profiling of LE (top) and OE (bottom). Full spectra (**A**) and extended spectral regions from δ 5.95 to 7.30 (**B**), from δ 3.2 to 5.5 (**C**), and from δ 3.1 to 0.7 (**D**). 1 = sucrose, 2 = *α*-glucose, 3 = *β*-glucose, 4 = citric acid, 5 = tyrosine, 6 = asparagine, 7 = fructose, 8 = malic acid, 9 = GABA, 10 = aspartic acid, 11 = succinic acid, 12 = proline, 13 = alanine.

In this work, the ^1^H NMR profiling of the extracts was important to have a picture of the primary metabolites, complementing the LC-DAD-ESI-MS analysis, which was focused on the secondary metabolites, whose concentration was too low to be detected through the ^1^H NMR profiling. The raw spectral data has been shared in a data repository [30]. Figure 1 shows the profiles elucidation, while Table 1 reported the results of the semi-quantitative analysis.

According to this analysis, LE and OE extracts were both rich in sugars, which is not surprising considering that they are by-products from fruit processing. In fact, the sugars comprised more than half of the extract mass (around 69% in LE and 88% in OE).

The most abundant sugar was fructose, yielding 318.1 ± 4.0 mg/g in LE and 347.6 ± 3.2 mg/g in OE, followed by glucose, whose overall concentration (including both α- and β-forms) was approximately 293 mg/g in LE and 305 mg/g in OE. Finally, sucrose was more abundant in OE (224.8 ± 2.3 mg/g) than LE (76.5 ± 1.0 mg/g). Conversely, as expected, citric acid was more abundant in LE (210.7 ± 4.6 mg/g) than OE (47.2 ± 1.0 mg/g). Both extracts contained GABA, while succinic acid and malic acid were found only in OE, and aspartic acid in LE. The profiles also revealed the presence of amino acids. Alanine and proline were detected in both extracts, while asparagine and tyrosine were detected only in LE and OE, respectivey.

#### 3.2.2. Secondary Metabolites: Phytochemical Screening and LC-DAD-ESI-MS Analysis

OE and LE secondary metabolites were firstly investigated by colorimetric assays aimed at quantifying the total phenolic compounds, flavonoids, flavan-3-ols and dihydrochalcones (vanillin index), as well as proanthocyanidins content (Table 2).

The quantification of these last two classes of compounds also allows calculation of the so-called polymerization index (vanillin index/proanthocyanidins), useful for determining whether an extract contains mainly monomeric or polymeric molecules. Indeed, proanthocyanidins are flavan-3-ols and/or flavan-3,4-diol oligomers, so that if the polymerization index is greater than 1, it indicates an abundance of monomeric molecules.

As shown in Table 2, OE and LE have comparable total phenolics and flavonoids content, while statistically significant differences (*p* < 0.01) were detected in terms of vanillin index and, therefore, in terms of concentration of monomeric molecules, which appear to be more present in LE rather than in OE. In any case, flavonoids appear to be the most abundant polyphenolic compounds in both extracts under examination as confirmed by subsequent phytochemical analyses carried out by LC-DAD-ESI-MS (Table 3).

Compounds were detected and tentatively identified by comparison of mass and UV–Vis spectra with literature data, online free consulting spectra databases as well as with commercially available reference standards (Table 3).

**Figure 2:**
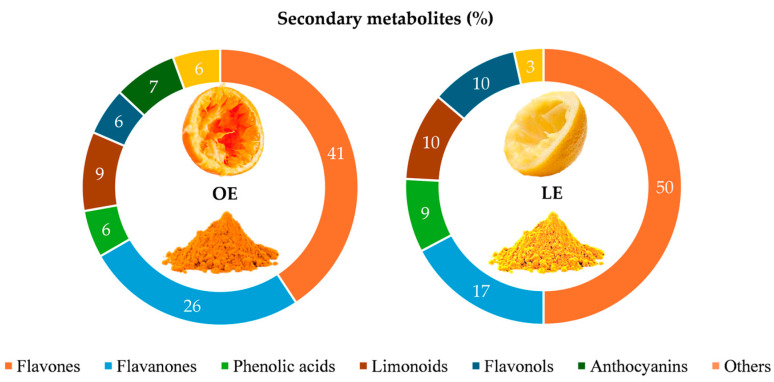
Distribution percentage of phytochemical classes identified in orange and lemon raw pomace extracts (OE and LE, respectively).

Eighty secondary metabolites have been identified (54 and 58 in OE and LE, respectively), belonging mainly to 6 classes: flavones (43%), flavanones (23%), phenolic acids (9%), limonoids (9%), flavonols (8%) and anthocyanins (5%). Of these, only 21 were common to OE and LE, showing a completely different phytochemical profile already from a qualitative point of view, as expected from two *Citrus* fruits belonging to different species. Indeed, as shown in Figure 2, although flavones were the most representative polyphenols class in both extracts under examination, they were mostly expressed in LE rather than OE (50% vs. 41%), whereas OE was characterized by a greater expression of flavanones (26% vs. 17% of LE). In addition, LE was also characterized, numerically, by the greatest content of phenolic acids, limonoids and flavonols (Figure 2). On the contrary, anthocyanins were detected only in OE, because it was obtained from raw pomace of blond oranges characterized by a light red streaks-pulp.

Numerically speaking, apigenin, kaempferol and diosmetin derivatives were the most abundant flavones, whereas among flavanones, the most representative compounds were eriodyctiol, naringenin and sakuranin derivatives.

However, the qualitative phytochemical profile, which sees flavones as predominant compounds, does not correspond to the quantitative phytochemical profile, which sees the clear predominance of flavanones, in particular hesperidin and narirutin in OE (2.36 ± 0.05 g/100 g and 0.37 ± 0.01 g/100 g, respectively), and hesperidin and eriocitrin in LE (1.20 ± 0.03 g/100 g and 1.14 ± 0.02 g/100 g, respectively).

### 3.3. Intestinal Bioaccessibility

**Figure 3:**
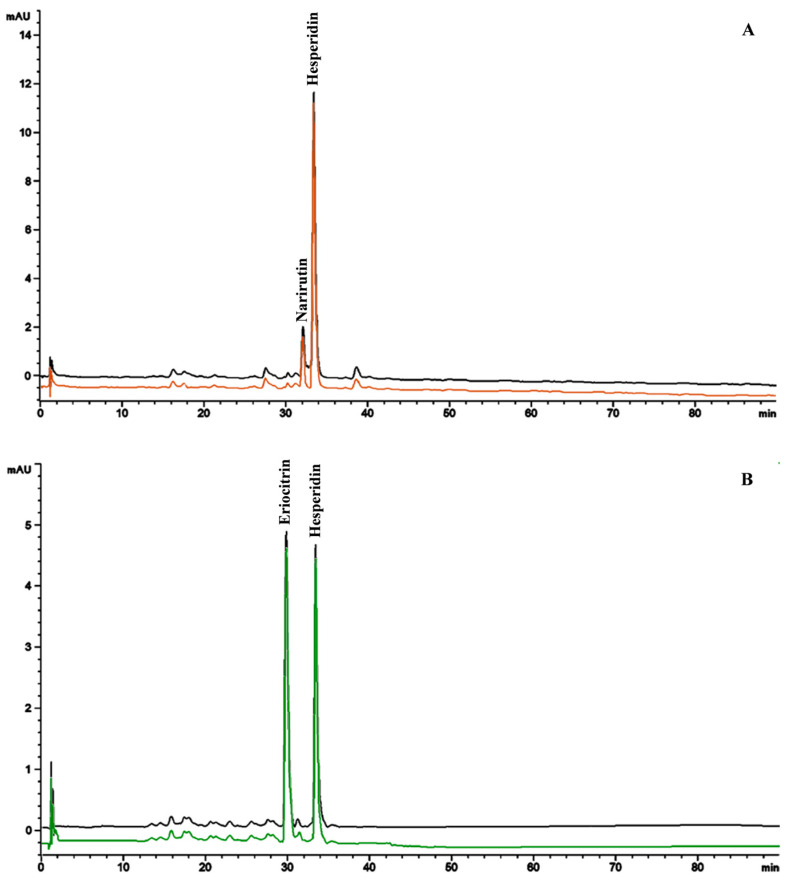
Representative LC-DAD chromatograms of orange raw pomace extract (OE, panel **A**) and lemon raw pomace extract (LE, panel **B**) pre- (black) and post-gastro-duodenal digestion (orange and green chromatogram, respectively) acquired at 292 nm.

To evaluate the bioaccessibility of the identified phytochemicals, OE and LE were subjected to a simulated in vitro gastro-duodenal digestion. The buccal digestion step was specifically skipped as the present study aimed to evaluate the bioaccessibility of the bioactive compounds within the extracts that will be potentially commercialized as a nutraceutical, therefore potentially formulated as tablets or caps. The aim was also to evaluate whether these extracts required also a gastro-resistant formulation to remain unchanged and thus exert their antioxidant and anti-inflammatory activity at the intestinal epithelium level. Quali-quantitative pre- and post-digestion analyses were carried out according to the validated LC-DAD-ESI-MS method described in Section 2.2.3. Results are shown in Figure 3.

No statistically significant difference was observed in the phytochemical profile of OE and LE between pre- and post-digestion analyses (Figure 3). These results were corroborated also by the quantification of the four most abundant compounds chosen as phytochemical markers (narirutin and hesperidin for OE, and eriocitrin and hesperidin for LE). Indeed, they showed comparable results between starting plant complexes (2.36 ± 0.05 g/100 g and 0.37 ± 0.01 g/100 g, for narirutin and hesperidin, respectively; and 1.20 ± 0.03 g/100 g and 1.14 ± 0.02 g/100 g, for eriocitrin and hesperidin, respectively) and relative digested samples (2.18 ± 0.07 g/100 g and 0.33 ± 0.02 g/100 g, for narirutin and hesperidin, respectively; and 1.14 ± 0.04 g/100 g and 1.08 ± 0.03 g/100 g, for eriocitrin and hesperidin, respectively), taking into account also the extraction process, which returned, during method validation, a recovery value ≥ 90%.

No interferences, such as any degradation products, metabolites, or co-eluting compounds, were recorded. Moreover, the chromatographic separation of the OE and LE constituents did not show any overlap or interferences from matrix constituents in the digested samples at the retention time of the identified phytochemicals, which appeared well-separated and easy identifiable.

### 3.4. Antioxidant and Anti-Inflammatory Activity

#### 3.4.1. In Vitro Cell-Free Assays

The antioxidant and anti-inflammatory activity of OE and LE was first investigated by in vitro spectrophotometric and spectrofluorimetric tests based on different environments and reaction mechanisms. This allowed evaluation of some specific activities such as the direct free-radical scavenging activity against several charged radicals, the iron-chelating capacity, the anti-peroxidative activity and the anti-inflammatory activity using enzymatic and non-enzymatic tests. Furthermore, this allowed us to make a first comparison between the two plant complexes and to establish which was the most appropriate range of concentrations to be tested in the Caco-2 cell model.

After a preliminary screening in a wide concentration range, four concentrations were selected for each extract with the aim of calculating the IC_50_ with the respective C.L. (Table 4).

Both extracts showed a similar trend, with a concentration-dependent antioxidant and anti-inflammatory behavior (R^2^ > 0.990) and the same order of potency: ORAC > BCB > TEAC > FRAP > DPPH for antioxidant assays, and PIA > ADA for anti-inflammatory assays. Despite the similar antioxidant and anti-inflammatory activity behavior of the two extracts, analyzing the IC_50_ values (Table 4), it is clear that, in accordance with the phytochemical data, LE, which is the richest in secondary metabolites, is also the strongest from both antioxidant and anti-inflammatory point of view, with statistically significant results in the DPPH (*p* < 0.001), BCB (*p* < 0.05) and ADA (*p* < 0.001) assays. Furthermore, according to the phytochemical data, it showed a significantly greater iron chelating capacity than OE, probably due to the conspicuous presence of monomeric molecules with free hydroxyl groups, mainly located in the ortho position, demonstrating, once again, how a linear correlation between secondary metabolites content and biological activity occurs.

#### 3.4.2. Effects of OE and LE on Cell Viability and Proliferation

**Figure 4:**
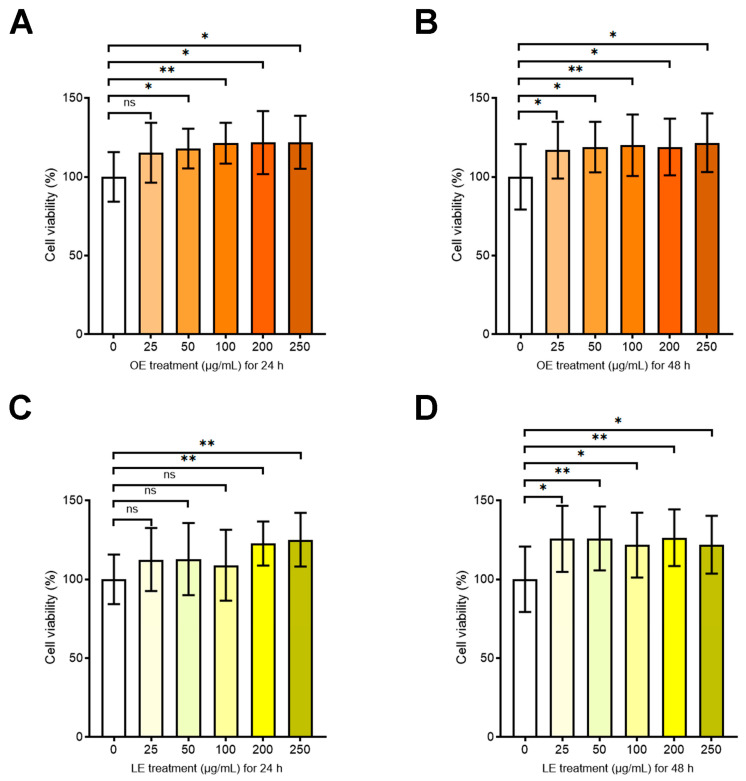
OE and LE effects on Caco-2 cell viability. Cell viability evaluated by XTT assay and expressed as percentage of cell viability in Caco-2 cells untreated or treated with different concentrations of OE for 24 h (**A**) and 48 h (**B**); and in Caco-2 cells untreated or treated with different concentrations of LE for 24 h (**C**) and 48 h (**D**). Values are the mean ± SD of three independent experiments repeated at least in quintuplicate. Data were analyzed by 2-tailed Student’s *t* test. * *p* < 0.05; ** *p* < 0.01; ns: non-significant.

To investigate the effects of OE and LE on an in vitro model of intestinal cells, we first evaluated the viability of Caco-2 cells after administration of OE and LE in the culture media at 25, 50, 100, 200, and 250 µg/mL for 24 h and 48 h. The highest DMSO concentration used was 0.1%. The results demonstrated that OE and LE had no significant cytotoxic effects; indeed, both compounds increased the cell viability with respect to the untreated control as determined by XTT assay (Figure 4). The effects of OE and LE on proliferation were also analyzed by cell confluence real-time monitoring by IncuCyte platform over a time course of 48h. In line with the increased cell viability, both OE and LE induced a more pronounced cell turnover with respect to the untreated control (Figure S1). No significant differences on cell viability and cell proliferation emerged between the different treatment concentrations, thus we chose to continue the subsequent experiments using the concentration closest to the average of the most promising IC_50_ values obtained by testing the extracts under examination (200 µg/mL for both OE and LE).

**Figure 5:**
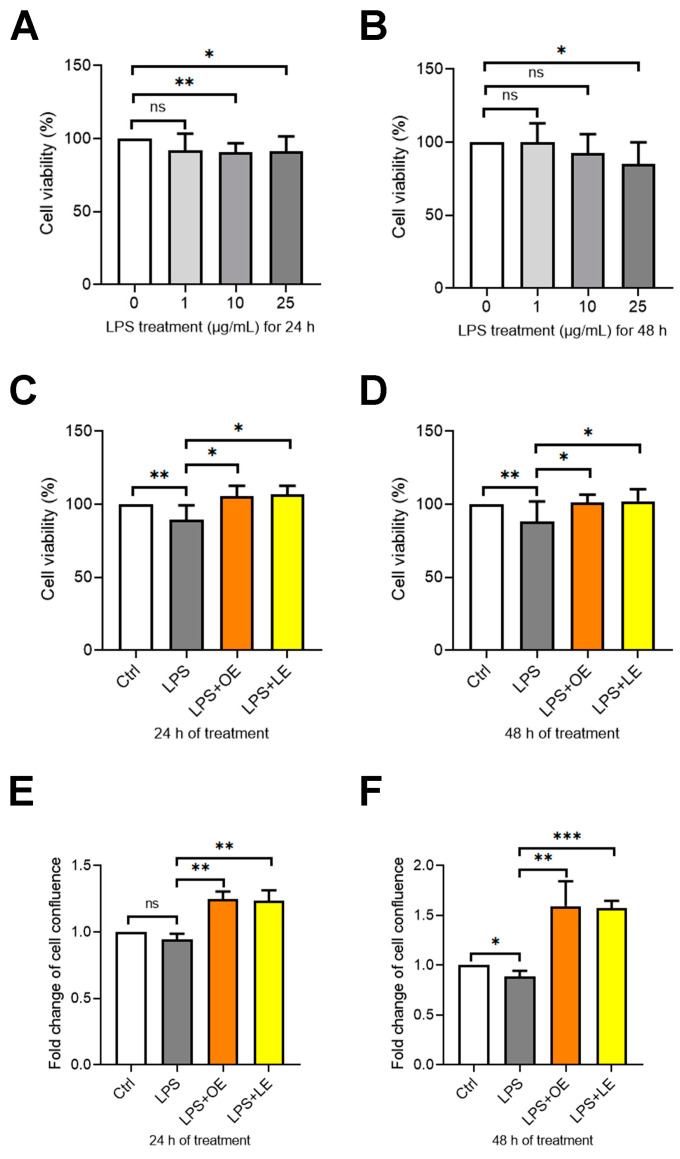
Cell viability and proliferation in Caco-2 cells under different treatments. Cell viability evaluated by XTT assay and expressed as percentage of cell viability in Caco-2 cells untreated or treated with different concentrations of LPS for 24 h (**A**) and 48 h (**B**); and in Caco-2 cells untreated (Ctrl) or treated with LPS, LPS + 200 µg/mL OE and LPS + 200 µg/mL LE for 24 h (**C**) and 48 h (**D**). Cell proliferation monitored by using the Incucyte live cell imaging system was expressed as fold change of mean cell confluence in Caco-2 cells Ctrl, LPS, LPS + 200 µg/mL OE, and LPS + 200 µg/mL LE for 24 h (**E**) and 48 h (**F**). Values are the mean ± SD of three independent experiments repeated at least in quintuplicate. Data were analyzed by 2-tailed Student’s *t* test. * *p* < 0.05; ** *p* < 0.01; *** *p* < 0.001; ns: non-significant.

Since previous studies reported that LPS stimulation was effective in inducing the typical damage occurring in IBD, including the disruption of the intestinal barrier, inflammatory and oxidant reactions [31,32], this model was established to assess the potential effects of OE and LE. Therefore, we treated Caco-2 cells with different concentrations of LPS (1, 10, and 25 µg/mL) to mimic the pathological condition. As shown in Figure 5A,B, LPS treatment (25 µg/mL) for 24 h and 48 h induced a maximum decrease of 15% in cell viability; thus this amount seemed to be the most suitable to induce the model without excessive cytotoxic effects. Then, the OE and LE ability to restore the cell viability and proliferation rate in LPS-treated Caco-2 cells, was investigated. As reported in Figure 5C–F, after 24 h and 48 h, the cell viability and confluency were significantly increased in LPS + OE and LPS + LE cells with respect to LPS-treated (LPS) or untreated cells (Ctrl).

#### 3.4.3. Effects of OE and LE on Intestinal Barrier Permeability

**Figure 6:**
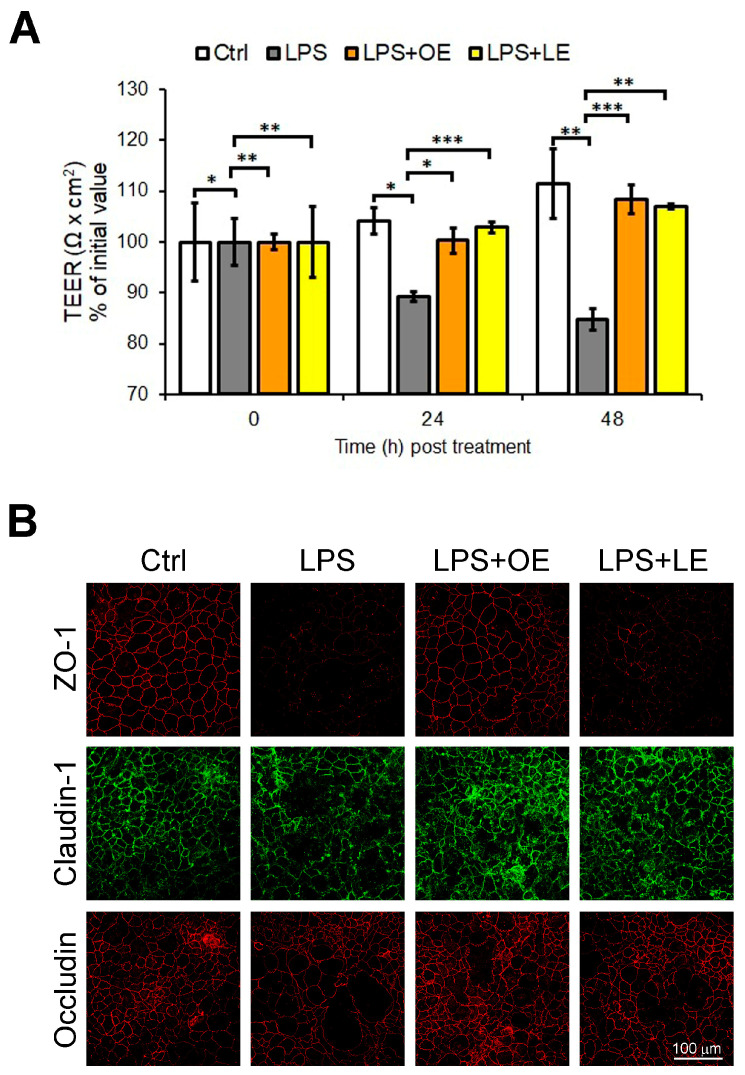
Intestinal permeability of Caco-2 cells under different treatments. (**A**) TEER values expressed as percentage of initial values of unit area resistance calculated by dividing resistance values by the effective membrane area in Caco-2 cells Ctrl, LPS, LPS + 200 µg/mL OE, and LPS + 200 µg/mL LE. Values are the mean ± SD of three independent experiments. Data were analyzed by 2-tailed Student’s *t* test. * *p* < 0.05; ** *p* < 0.01; *** *p* < 0.001. (**B**) Representative immunofluorescence by confocal imaging of ZO-1, Claudin-1, and Occludin in Caco-2 cells Ctrl, LPS, LPS + 200 µg/mL OE, and LPS + 200 µg/mL LE. 40× magnification.

We next sought to analyze the integrity of Caco-2 cell monolayers after 24 h and 48 h treatment with LPS, LPS + 200 µg/mL OE and LPS + 200 µg/mL LE. Our data revealed that after 24 h of treatment, LPS induced a significant decrease of TEER mean values, an effect that was intensified after 48 h (Figure 6A). On the contrary, as shown in Figure 6A, both OE and LE were able to counteract the effect of LPS by maintaining the TEER mean values near to the control untreated cells (Ctrl). To confirm this functional effect, the expression of the tight junction (TJ) proteins ZO-1, Claudin-1, and Occludin by immunofluorescence staining was also evaluated. As shown in Figure 6B, 48 h LPS treatment caused a decreased expression of TJ proteins, but this reduction was less evident in LPS + 200 µg/mL OE and LPS + 200 µg/mL LE, especially under OE treatment.

#### 3.4.4. Effects of OE and LE on Oxidative Stress and Inflammatory Response

**Figure 7:**
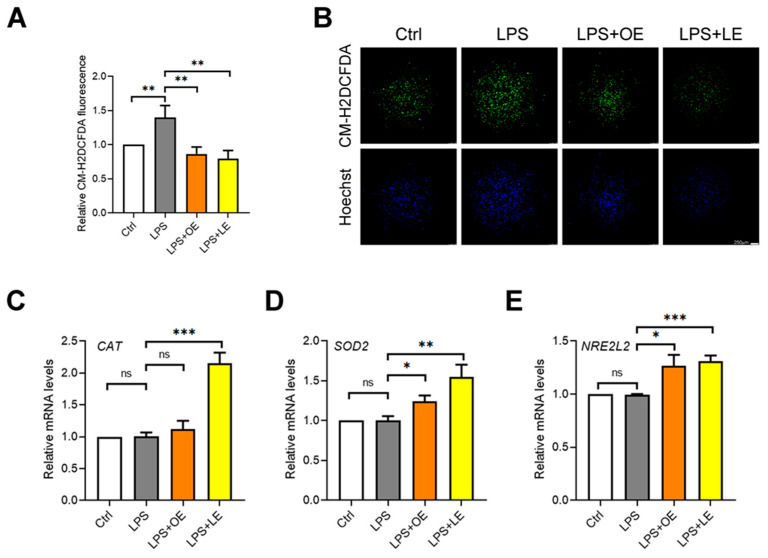
Oxidative stress of Caco-2 cells under different treatments. Fold change of the relative mean fluorescence (**A**) and representative images (**B**) of CM-H2DCFDA (green) staining in Caco-2 cells Ctrl, LPS, LPS + 200 µg/mL OE, and LPS + 200 µg/mL LE. Hoechst nuclear staining (blue). 40× magnification. Relative mRNA expression of CAT (**C**), SOD2 (**D**), and NRE2L2 (**E**) genes measured by qPCR in Caco-2 cells Ctrl, LPS, LPS + 200 µg/mL OE, and LPS + 200 µg/mL LE. Values are the mean ± SD of three independent experiments. Data were analyzed by 2-tailed Student’s *t* test. * *p* < 0.05; ** *p* < 0.01; *** *p* < 0.001; ns: non-significant.

To evaluate the potential antioxidant effect of OE and LE in the Caco-2 cells model resembling the impairment of intestinal permeability (i.e., LPS treatment), the intracellular ROS levels as well as the gene expression levels of the antioxidant enzymes *CAT*, *SOD2* and *NRE2L2* (gene encoding for Nrf2), were evaluated. The CM-H_2_DCFDA-staining revealed that, after 4 h, both OE and LE significantly reduced the rate of increase of LPS-dependent ROS levels in Caco-2 cells (Figure 7A,B). Moreover, as reported in Figure 7C–E, even if after 24 h LPS treatment was ineffective on the expression of *CAT*, *SOD2* and *NRE2L2* genes with respect to the control cells, the addition of 200 µg/mL OE or LE caused the up-regulation of all the antioxidant genes.

**Figure 8:**
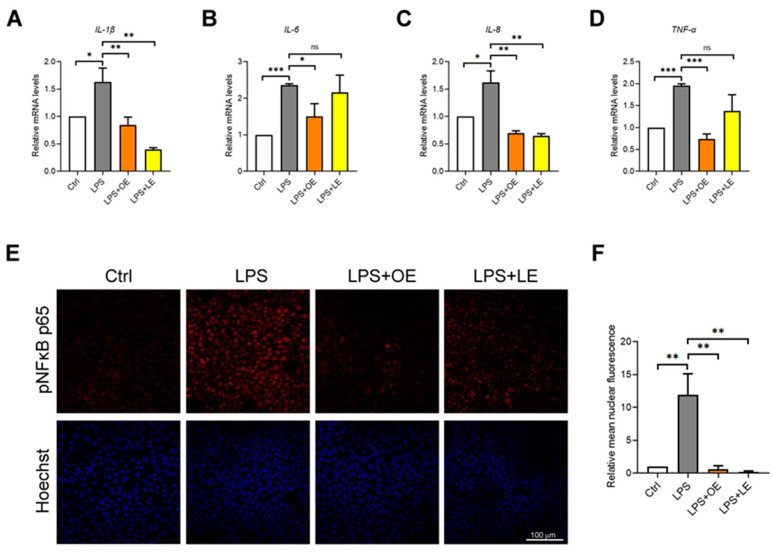
Inflammatory response of Caco-2 cells under different treatments. Relative mRNA expression of *IL-1β* (**A**), *IL-6* (**B**), *IL-8* (**C**), and *TNF-α* (**D**) genes measured by qPCR in Caco-2 cells Ctrl, LPS, LPS + 200 µg/mL OE, and LPS + 200 µg/mL LE. Representative immunofluorescence by confocal imaging (**E**) and QFIA (**F**) of pNFκB p65 (red) in Caco-2 cells Ctrl, LPS, LPS + 200 µg/mL OE, and LPS + 200 µg/mL LE. Hoechst nuclear staining (blue). 40× magnification. Values are the mean ± SD of three independent experiments. Data were analyzed by 2-tailed Student’s *t* test. * *p* < 0.05; ** *p* < 0.01; *** *p* < 0.001; ns: non-significant.

Finally, the effects on inflammatory response after treatments were assessed. As shown in Figure 8A–D, LPS induced a significant increase in gene expression of the pro-inflammatory cytokines IL-1β, IL-6, IL-8, and TNF-α, while 200 µg/mL OE and 200 µg/mL LE prevented this effect. According to the increased pro-inflammatory genes, LPS treatment also enhanced nuclear translocation of the phosphorylated/active form of nuclear factor kappa-light-chain-enhancer of activated B cells p65 (pNFκB p65), but this effect was not observed when 200 µg/mL OE and 200 µg/mL LE were added to LPS-treated cells (Figure 8E,F).

## 4. Discussion

Every year, it is estimated that about 15 million tons of *Citrus* by-products are produced worldwide [33]. However, the chemical composition of *Citrus* by-products, as can be expected from any vegetable raw material, changes depending on the pedo-climatic conditions to which the native plant is exposed, and on the fruit processing (e.g., to obtain juice or essential oil) and extraction method applied to recover the phytochemicals of interest [34]. Considering this, the critical steps to be addressed in preparing plant complexes to be used in the nutraceutical and pharmaceutical field are the selection of the most appropriate green extraction technique, its optimization and standardization, an in-depth characterization of the obtained extracts, their titration, bioaccessibility studies as well as the evaluation of the health properties by pre-clinical studies [35]. Once the starting material has been selected, the extraction technique and conditions must be optimized, not only in terms of the extracted compounds, but also in terms of phytochemical profile. Indeed, *Citrus* raw pomace contains different phytochemicals with powerful bioactivities that can potentially find application in the nutraceutical and pharmaceutical fields, especially in the context of chronic inflammatory diseases.

Despite being a waste product, *Citrus* pomace represents one of the major sources of polyphenols as the latter are mainly distributed in the flavedo and albedo, rather than in the edible part of the fruit. Considering this, one of the main limiting factors for *Citrus* agro-industrial residue utilization is the lack of a cost-effective extraction method for high-quality compounds. Green extractions have the potential to overcome such limitations and provide higher yields and energy savings [36].

The main goal of a green method is to avoid the use of toxic solvents. Over the years, several supercritical fluids and ionic liquids have been investigated. The former, however, are too expensive for industrial scalability and too selective for lipophilic compounds, while the use of the ionic liquids is rather controversial because they seem to be potentially harmful for the environmental eco-system. Considering this, the most cheap and renewable solvents remain ethanol and water, and the limiting factor becomes the extraction technique used. Putnik and co-workers [35] reviewed the latest studies concerning novel and greener methods for valorization of *Citrus* by-products. Microwaves, ultrasound, pulsed electric fields and high-pressure methods were compared between themselves and to the conventional techniques to highlight pros, cons and potential scalability of these technologies. Ultrasound-assisted extraction, disrupting cells by cavitation and promoting the diffusion of bioactive compounds from plant matrix via solvents, has proved the most cheap, reproducible and simple alternative to conventional extraction methods for the recovery of bioactive compounds from *Citrus* raw pomace. Furthermore, it gives higher extraction yields at lower temperatures and extraction times, ideal parameters for photosensitive and thermolabile compounds [35]. Several authors have recently evaluated and optimized the ultrasound exposure, solvent type, and solvent concentration for the extraction of polyphenols from *Citrus* pomace, and the best yields were achieved, according to our results, with hydroalcoholic mixture with 80% organic solvent, using a matrix/solvent ratio of 1:10, *w*/*v* [37,38].

It is well-known that *Citrus* pomace is a rich source of polyphenols (0.91–4.92%), with flavonoids accounting for 2–3% [39,40]. According to our results, several subclasses of flavonoids have been detected in *Citrus* pomace. Naringenin, hesperetin, narirutin, naringin, hesperidin, neohesperidin, eriocitrin, neoeriocitrin, poncirin, and didymin were the main identified flavanones and flavanonols [41]. Apigenin-6,8-di-*C*-glucoside, apigenin-7-*O*-rutinoside, diosmin, chrysoeriol-*C*-glucoside, rutin, and kaempferol-3-*O*-rutinoside [42] were the most abundant flavones and flavonols. Finally, poly-methoxylated-flavones such as nobiletin and tangeretin [43,44], as well as prothocyanidins and anthocyanins [45], were previously detected. In orange and lemon raw pomace, in line with our results, hesperidin, narirutin and eriocitrin are always the most abundant flavonoids detected [35]. Other minor previously detected compounds in *Citrus* pomace were phenolic acids, such as ferulic, caffeic and sinapic acid [46], and limonoids such as limonin, nomilin, ichangin and derivatives [47].

In addition, several primary metabolites, including simple sugars, amino acids and organic acids, were detected in *Citrus* raw pomace. In the early stage of fruit development, sucrose is the major accumulated sugar, with a sucrose–glucose–fructose ratio of 2:1:1 [47]. However, during fruit maturation, it is hydrolyzed either to fructose and UDP-glucose by sucrose synthase, or to glucose and fructose by invertase. Accordingly, we observed that the ratios between sugars changed in favor of glucose and fructose.

Although citrate is the major organic acid accumulated in *Citrus* fruit, the synthesis and accumulation of a discrete amount of malic acid in orange and lemon fruit were already reported [48], whereas other organic acids such as oxalic, tartaric, benzoic, succinic, and malonic were detected only in traces [49].

Aspartic acid, asparagine, proline and GABA have been previously detected among the most abundant amino acids in *Citrus* fruit. Generally, they increase during fruit maturation; however, a conspicuous difference in terms of total amino acids content between different *Citrus* species was observed, with lemon showing, in line with our results, a higher content with respect to sweet orange [50].

Many studies have shown that *Citrus* extracts decrease the onset and progression of several chronic diseases by preventing oxidative stress, tissue damage, and inflammatory processes [51,52,53].

The comparison of the radical scavenging activity measured by in vitro assays based on different mechanisms and reaction environments allows the establishment of key structure–activity relationships (SAR). Recently, it has been demonstrated how hesperidin, hesperetin and neohesperidin were found to be more active in hydrogen atom transfer assays such as ORAC and TEAC, whereas eriocitrin and neoeriocitrin were more active in electron transfer assays, such as FRAP and DPPH. Furthermore, it has been also demonstrated that by combining them, they showed an interesting synergistic antioxidant activity [10]. These results were also corroborated by the evaluation of the anti-inflammatory activity, investigated by the same assays carried out in the present study (ADA and PIA), where the flavanones’ mix showed the strongest anti-inflammatory activity [10]. These data appear even more interesting considering the number of bioactive compounds present in a plant complex and the ability of flavanones to remain unchanged, after in vitro simulated gastro-duodenal digestion [10], and after 12 and 24 h in the small intestine and in the colon of rats after oral administration of a *Citrus* extract [54].

These properties are directly correlated to the flavonoids content of *Citrus* fruit, able to inhibit different enzymes involved in different cellular processes [55], but also to minor compounds such as phenolic acids and limonoids with well-known strong free radical quenching activity [46].

Here, we investigated the biological effects of OE and LE in an in vitro model of IBD consisting of Caco-2 cell monolayers stimulated with LPS to induce the typical damage observed in this disease. In our model, 25 µg/mL LPS treatment, even if continued for 48 h, did not cause strong toxicity but reduced cell viability and proliferation by approximately 15%. Accordingly, other studies demonstrated that LPS may act on Caco-2 cells as an antiproliferative and inflammatory stimulus able to impair gut barrier integrity [56,57]. On the other hand, other authors reported that LPS may upregulate cell proliferation rates [58,59]. This discrepancy may be attributable to the source of the LPS used. Indeed, LPS structure is variable between different bacterial strains, and this can influence its effects [60]. In any case, the evidence that LPS, regardless of bacterial strain, can destroy the integrity of the intestinal barrier through disruption of TJs is numerous, as is the evidence that various compounds can play a protective role in this process [61]. The present in vitro results seem to confirm the beneficial effects of orange and lemon raw pomace. Indeed, our findings highlighted that OE and LE were able to counteract the LPS detrimental effect on cell proliferation and intestinal barrier integrity. Accordingly, it has been demonstrated that hesperidin enhances the intestinal barrier integrity in Caco-2 cell monolayers increasing the TEER as well as the mRNA expression and protein levels of occludin, MarvelD3, JAM-1, claudin-1, and claudin-4 [62]. A similar effect was reported also in Caco-2 and a RAW264.7 cells co-culture model treated with naringenin, nobiletin and hesperetin [63]. The protective effect of OE and LE against the LPS-dependent impairment of intestinal barrier integrity and TJ destruction, as well as for other natural compounds, could be mechanistically associated to the activity of these plant complexes on oxidative stress and inflammation [64,65]. Indeed, our data demonstrated that OE and LE may suppress ROS production, thus hampering the vicious cycle between NFkB p65 nuclear translocation and consequent transcriptional activation of pro-inflammatory and repression of antioxidant genes.

These effects confirm the evidence that flavanones and polymethoxylated flavones are inhibitors of important proteins involved in the activation of the inflammatory cascade [22,66]. It has been demonstrated that hesperidin was able to inhibit the mitogen-activated protein kinases (MAPKs) and phosphodiesterases [67], whereas several flavanones were found to down-regulate NFκB [68,69], in turn involved in the modulation of *iNOS*, *COX-2*, *IL-6*, and *TNF-α* gene expression [70].

We have also previously demonstrated that hesperidin, neohesperidin, hesperetin, eriocitrin, and eriocitrin exhibit strong antioxidant activity by reducing the ROS release, the formation of carbonylated proteins and lipid peroxides, as well as the oxidation of GSH to GSSG in Caco-2 cell monolayers. They were also able to exert strong anti-inflammatory activity by inhibiting COX enzymes, with a selectivity towards COX-2, as also demonstrated by molecular modelling studies [22]. Indeed, all these factors may contribute to the evident beneficial effects that we found under OE and LE treatments. Several studies tested other natural compounds against IBD [63], but among them, only few compounds exhibit as broad a range of effects as we have seen in the present study. 

In summary, in the present study, we found that both OE and LE preserved the integrity of the intestinal barrier against LPS-induced damage due to colonization of pathogenic bacteria [71]. However, another important aspect to consider is that in IBD, intestinal barrier dysregulation alone is insufficient to cause disease, but enhanced gut permeability can also accelerate disease onset and increase severity by the activation of pro-inflammatory ROS-sensitive pathways in immune cells [72]. Therefore, although this hypothesis requires further experimental studies to be confirmed, it is conceivable that OE and LE may also counteract intestinal dysbiosis, thus representing a promising therapeutic approach to reverse the IBD exacerbation. 

## 5. Conclusions

In conclusion, this study demonstrated that orange and lemon raw pomace may be considered for the development of drugs and nutraceutical products for the treatment and prevention of IBD. The combination of a wide range of substances such as flavones, flavanones, phenolic acids, limonoids, etc. confer on them a potentially high therapeutic effectiveness on the gut barrier, acting via different mechanisms that include preservation of TJ proteins and activation of the antioxidant and anti-inflammatory pathways. The possibility of overcoming the high cost of processing waste is also a strong advantage.

However, being this a preliminary study based on in vitro cell-free and cell-based models, the results transability to the complex in vivo scenario must be done very carefully. Therefore, further in vivo and clinical studies to deeply investigate the antioxidant and anti-inflammatory properties of these plant complexes, as well as the molecular mechanisms and cellular targets involved, are needed to justify their potential role in IBD management.

## Figures and Tables

**Table 1:** Semi-quantitative analysis done by ^1^H NMR of the compounds identified in orange and lemon raw pomace extracts (OE and LE). Results are expressed in mg metabolite/g of dried extract (DE) and each value is the mean ± standard deviation of five independent measurements.

Metabolite	Diagnostic Signal (δ, Multiplicity *)	LE (mg/g DW)	OE (mg/g DW)
alanine	1.45, d	3.6 ± 0.1	1.9 ± 0.1
α-glucose	5.20, d	109.3 ± 1.4	108.2 ± 1.0
β-glucose	4.6, d	183.6 ± 2.4	197.5 ± 2.0
sucrose	5.4, d	76.5 ± 1.0	224.8 ± 2.3
fructose	3.87, dd	318.1 ± 4.0	347.6 ± 3.2
citric acid	2.71, d	210.7 ± 4.6	47.2 ± 1.0
proline	2.09, m	8.0 ± 0.1	12.9 ± 0.3
GABA	3.02, t	2.5 ± 0.1	4.42 ± 0.1
asparagine	2.95, dd	21.5 ± 1.2	n.d. ^§^
aspartic acid	2.6, dd	23.9 ± 0.7	n.d.
succinic acid	2.47, s	n.d.	0.4 ± 0.1
tyrosine	7.28, d	n.d.	3.1 ± 0.1
malic acid	4.3, dd	n.d.	15.7 ± 0.1

* d = doublet, dd = double doublet, m = multiplet, t = triplet, s = singlet; ^§^ n.d. = not detected.

**Table 2:** Phytochemical screening of orange and lemon raw pomace extracts (OE and LE). Results are the mean ± standard deviation (S.D.) of three independent experiments in triplicate (*n* = 3).

Phytochemical Assay	OE	LE
Total phenols (g GAE ^a^/100 g DE ^b^)	2.41 ± 0.16	2.46 ± 0.14
Flavonoids (g RE ^c^/100 g DE)	1.36 ± 0.09	1.53 ± 0.08
Vanillin index (mg CE ^d^/100 g DE)	0.36 ± 0.01	0.46 ± 0.02 **
Proanthocyanidins (mg CyE ^e^/100 g DE)	0.003 ± 0.000	0.004 ± 0.000
Polymerization index ^f^	139.17	111.41 **

^a^ GAE, gallic acid equivalents; ^b^ DE, dry extract; ^c^ RE, rutin equivalents; ^d^ CE, Catechin equivalents; ^e^ CyE, Cyanidin equivalents; ^f^ Polymerization index = vanillin index/proanthocyanidins. ** *p* < 0.01 vs. OE.

**Table 3:** Secondary metabolites of orange and lemon raw pomace extracts (OE and LE, respectively) tentatively identified by LC-DAD-ESI-MS using both the positive and negative ionization modes.

Compound Name	RT ^b^ (min)	Molecular Formula	Molecular Weight	[M−H]^−^ (*m*/*z*)	[M+H]^+^ (*m*/*z*)	OE ^c^	LE ^d^
6-Hydroxyapigenin (Scutellarein) ^a^	16.1	C_15_H_10_O_6_	286		287	−	+
Luteolin-8-glucoside (Orientin) ^a^	16.2	C_21_H_20_O_11_	448	447		−	+
Apigenin 6-*C*-glucoside 8-*C*-arabinoside	16.8	C_26_H_28_O_14_	564		565	+	−
Kaempferol 7-*O*-glucoside ^a^	17.4	C_21_H_20_O_11_	448		449	−	+
Dihydroferulic acid 4-*O*-glucuronide	17.5	C_16_H_20_O_10_	372	371		−	+
Dihydrocaffeic acid dimer	18.7	C_18_H_20_O_8_	364		365	−	+
Heptyl caffeate	19.4	C_16_H_22_O_4_	278	277		−	+
Feruloylisocitric acid	19.6	C_16_H_16_O_10_	368	367		+	−
Naringenin 7-*O*-glucoside ^a^	19.8	C_21_H_22_O_10_	434	433		+	−
Hydroxycaffeic acid	20.0	C_9_H_8_O_5_	196		197	+	−
Apigenin 7-*O*-rutinoside ^a^	20.4	C_27_H_30_O_14_	578		579	+	+
Apigenin 7,4′-diglucoside	21.4	C_27_H_30_O_15_	594		595	−	+
Diosmetin 3′-*O*-glucuronide	21.7	C_22_H_20_O_12_	476	475		+	−
Quercetin 3-*O*-galactoside (Hyperoside) ^a^	22.6	C_21_H_20_O_12_	464	463		−	+
Sakuranin	22.7	C_22_H_24_O_10_	448		449	+	+
Diosmetin 6,8-di-*C*-glucoside	23.2	C_28_H_32_O_16_	624		625	+	+
Diosmetin-7-*O*-glucoside ^a^	23.4	C_22_H_22_O_11_	462		463	−	+
Chrysoeriol-*C*-glucoside	25.1	C_22_H_22_O_11_	462		463	−	+
Kaempferol 3-*O*-rhamnoside ^a^	25.2	C_21_H_20_O_10_	432		433	+	−
Quercetin 3-rutinoside (Rutin) ^a^	26.4	C_27_H_30_O_16_	610		611	+	+
Apigenin 6,8-C-diglucoside (Vicenin 2)	27.4	C_27_H_30_O_15_	594		595	+	−
Isosakuranetin-7-*O*-rutinoside (Didymin) ^a^	28.0	C_28_H_34_O_14_	594	593	595	+	+
Kaempferol-3-*O*-rutinoside ^a^	28.4	C_27_H_30_O_15_	594		595	+	−
Perilloside A	28.9	C_16_H_26_O_6_	314	313		−	+
Isorhamnetin 3-*O*-rutinoside (Narcissin) ^a^	29.0	C_28_H_32_O_16_	624	623	625	−	+
Hesperetin-glucuronide-sulfate	29.2	C_22_H_22_O_15_S	558		559	+	−
Quercetin-3-*O*-sophoroside (Baimaside)	29.4	C_27_H_30_O_17_	626	625		−	+
Limonin glucoside	29.9	C_32_H_42_O_14_	650	649		+	−
Sinigrin	30.0	C_10_H_16_KNO_9_S_2_	397	396		+	−
Eriodictyol-7-*O*-rutinoside (Eriocitrin) ^a^	30.2	C_27_H_32_O_15_	596	595	597	−	+
3-Hydroxyphloretin 2′-*O*-xylosyl-glucoside	30.5	C_26_H_32_O_15_	584		585	+	−
Eriodictyol 7-*O*-neohesperidoside (Neoeriocitrin) ^a^	31.2	C_27_H_32_O_15_	596	595	597	−	+
Diosmetin-7-O-rutinoside (Diosmin) ^a^	31.4	C_28_H_32_O_15_	608	607	609	+	+
5,3′,4′-Trihydroxy-3-methoxy-6,7-methylenedioxyflavone 4′-*O*-glucuronide	31.9	C_23_H_20_O_14_	520	519		+	−
Hesperetin-3′,5,7-tri-sulfate	32.1	C_16_H_14_O_14_S_3_	525		526	+	−
Limocitrin *O*-3-hdroxy-3-methylglutaryl (HMG)-glucoside	32.2	C_29_H_32_O_17_	652		653	+	+
Narirutin ^a^	32.4	C_27_H_32_O_14_	580	579		+	−
Apigenin 7,4′-diglucoside	32.9	C_27_H_30_O_15_	594	593		−	+
Apigenin 7-sulfate	33.0	C_15_H_10_O_8_S	350		351	+	−
Limonin	33.2	C_26_H_30_O_8_	470		471	−	+
Hesperetin-7-rutinoside (Hesperidin) ^a^	33.7	C_28_H_34_O_15_	610	609	611	+	+
Hesperetin-7-neohesperidoside (Neohesperidin) ^a^	34.6	C_28_H_34_O_15_	610	609	611	+	+
Naringenin hexosyl-deoxyhexoside	35.0	C_27_H_32_O_14_	580		581	−	+
Methyl-limonexic acid	35.6	C_27_H_32_O_10_	516		517	+	−
Diosmetin-7-O-neohesperidoside (Neodiosmin) ^a^	35.9	C_28_H_32_O_15_	608	607	609	−	+
Kaempferol 3-*O*-(6″-acetyl-galactoside) 7-*O*-rhamnoside	36.1	C_29_H_32_O_16_	636		637	+	−
Nomilin hexoside	36.4	C_34_H_46_O_15_	694	693		+	+
Quercetin 3-rhamnoside (Quercitrin) ^a^	36.6	C_21_H_20_O_11_	448	447	449	+	+
Nomilinic acid-O-hexoside	38.2	C_34_H_48_O_16_	712	711		+	+
Pelargonidin 3-O-(6″-succinyl-glucoside)	38.7	C_25_H_24_O_13_	533		534	+	−
Isosakuranetin-7-O-neohesperidoside (Poncirin) ^a^	39.2	C_28_H_34_O_14_	594	593		+	−
Cyanidin 3-O-xylosyl-rutinoside	41.8	C_32_H_38_O_19_	727	726	728	+	−
Kaempferol *O*-synapoyl-caffeoyl-sophoroside-*O*-hexoside	42.9	C_53_H_56_O_28_	1141	1139		+	−
Kaempferol-isorhamninoside-rhamnoside	43.7	C_39_H_50_O_23_	886		887	+	+
Ichangin	44.5	C_26_H_32_O_9_	488	487		+	−
Acacetin (di-deoxyhexosyl)-hexoside	45.7	C_34_H_42_O_18_	738	737		+	−
Hydroxy-pentamethoxyflavanone (Norcitromitin)	46.2	C_20_H_22_O_8_	390	389	391	+	−
Naringin ^a^	47.6	C_27_H_32_O_14_	580		581	+	+
Feruloylquinic acid	48.6	C_17_H_20_O_9_	368		369	+	+
4′,5,6,7,8-Pentamethoxyflavone (Tangeretin) ^a^	49.3	C_20_H_20_O_7_	372		373	+	−
Nomilinic acid	50.0	C_28_H_36_O_10_	532	531		+	+
Peonidin 3-*O*-glicoside	51.2	C_22_H_22_O_11_	462		463	+	−
Hesperetin-7-sulfate-3′,5-di-glucuronide	52.1	C_28_H_26_O_20_S	718		719	+	−
6-Demethoxytangeretin	55.2	C_19_H_18_O_6_	342		343	+	+
Sinapic acid ^a^	55.3	C_11_H_12_O_5_	224	223		−	+
Pelargonidin 3-*O*-sambubioside	56.4	C_26_H_28_O_14_	565		566	+	−
3,7-Di-*O*-methylquercetin	56.5	C_17_H_14_O_7_	330		331	−	+
Eriodictyol ^a^	58.1	C_15_H_12_O_6_	288		289	+	+
Deacetylnomilin	58.8	C_26_H_32_O_8_	472		473	−	+
4′-*O*-Methylkaempferol (Kaempferide) ^a^	62.0	C_16_H_12_O_6_	300		301	+	+
5,6-Dihydroxy-7,8,3′,4′-tetramethoxyflavone (Pebrellin)	63.6	C_19_H_18_O_8_	374		375	+	−
Homoeriodictyol chalcone ^a^	63.7	C_16_H_14_O_6_	302		303	−	+
Desmethyltangeretin (Gardenin B)	66.1	C_19_H_18_O_7_	358		359	+	+
Desmethylnobiletin	66.4	C_20_H_20_O_8_	388		389	−	+
4′,5-dihydroxy-6,7,8-trimethoxyflavone (Xanthomicrol)	70.6	C_18_H_16_O_7_	344	343		−	+
Naringenin-sulfate	71.2	C_15_H_10_O_9_S	366		367	+	−
Citrusin III	71.4	C_36_H_53_N_7_O_9_	727		728	−	+
5-*O*-Methylmikanin	75.0	C_19_H_18_O_7_	358		359	+	+
Kaempferol 3,5-dimethyl ether	80.3	C_17_H_14_O_6_	314		315	+	+
Naringenin-4′-methylether (Isosakuranetin) ^a^	80.6	C_16_H_14_O_5_	286	285		+	+

^a^ Check with commercially available HPLC-grade reference standards (purity ≥ 98%, Extrasynthase, Genay, France); ^b^ RT, retention time; ^c^ OE, orange raw pomace dry extract; ^d^ LE, lemon raw pomace dry extract; −, absent, +, present.

**Table 4:** Antioxidant and anti-inflammatory activity of orange and lemon raw pomace extracts (OE and LE) in comparison with the reference standards. Results, which represent the mean of three independent experiments in triplicate (*n* = 3), are expressed as g of reference standard equivalents (RSE)/100 g dry extract (DE), and as the concentration inhibiting 50% of the oxidant/inflammatory activity (IC_50_) with 95% confidence limits (between brackets).

Test	OE	LE	OE	LE	RS
	g RSE ^a^/100 g DE	g RSE ^a^/100 g DE		IC_50_ (µg/mL)	
DPPH	0.77 ± 0.10 ***	1.61 ± 0.16	3810.09 (2231.51–4505.38) ***	1015.23 (803.66–1282.50)	11.62 (9.82–13.75)
TEAC	3.95 ± 0.29	2.44 ± 0.45	127.26 (105.28–153.81)	182.49 (152.76–218.00)	3.73 (1.51–9.24)
FRAP	1.80 ± 0.09	1.71 ± 0.12	119.80 (112.66–354.32)	199.15 (119.14–332.89)	3.68 (1.61–8.46)
ORAC	15.13 ± 1.10	15.19 ± 0.22	7.36 (5.81–9.34)	5.98 (4.93–7.25)	0.67 (0.20–1.16)
BCB	0.82 ± 0.03 **	1.33 ± 0.05	76.65 (54.72–107.36) *	41.60 (32.68–52.96)	0.32 (0.15–0.55)
ICA	39.92 ± 3.01 ***	50.27 ± 0.58	2140.12 (1329.71–3444.44) ***	535.88 (424.92–675.82)	5.65 (2.50–7.75)
ADA	2.28 ± 0.21 ***	11.29 ± 1.01	3825.66 (2961.86–4941.34) ***	785.09 (188.93–1362.44)	29.67 (17.56–50.14)
PIA	79.63 ± 1.77 **	92.17 ± 4.88	210.15 (184.46–239.41)	151.14 (95.83–238.38)	28.75 (14.41–57.34)

^a^ RSE, Reference standard equivalents: Trolox for FRAP, DPPH, TEAC, and ORAC assay; BHT for β-carotene bleaching assay; diclofenac sodium for anti-inflammatory assays (ADA and PIA); * *p* < 0.05 vs. LE; ** *p* < 0.01 vs. LE *** *p* < 0.001 vs. LE.

## Data Availability

The original contributions presented in the study are included in the article/Supplementary Materials, further inquiries can be directed to the corresponding author.

## Supplementary Materials

The following supporting information can be downloaded at: https://www.mdpi.com/article/10.3390/antiox13070869/s1, Table S1: List of antibodies used; Figure S1: OE and LE effects on Caco-2 cell proliferation.
