# Supplementary material for: Citrus Pomace as a Source of Plant Complexes to Be Used in the Nutraceutical Field of Intestinal Inflammation
Source: Antioxidants (Basel). 2024 Jul 19;13(7):869. doi: 10.3390/antiox13070869 (PMC11274116; doi:10.3390/antiox13070869)

Supplementary Material

Citrus pomace as a source of plant-complexes to be used in the nutraceutical field of intestinal inflammation

Mariarosaria Ingegneri [1,§], Maria Rita Braghini [2,§], Michela Piccione[3], Cristiano De Stefanis[3], Manuela Mandrone[4], Ilaria Chiocchio[4], Ferruccio Poli[4], Martina Imbesi[1], Antonella Smeriglio[1,*], Anna Alisi[2,*], Domenico Trombetta[1]

Table S1. List of antibodies used.

Protein target	Source	Catalogue number
ZO-1	Invitrogen	40-2200
Claudin-1	Invitrogen	37-4900
Occludin	Invitrogen	40-4700
Nrf2	Santa Cruz Biotechnology	sc-722
Phospho-NF-κB p65 (Ser536)	Cell Signaling Technology	3033
Alexa Fluor 488	Invitrogen	A-11017
Alexa Fluor 555	Invitrogen	A-21430

Figure S1. OE and LE effects on Caco-2 cell proliferation. Cell proliferation monitored by using the Incucyte live cell imaging system and expressed as fold change of mean cell confluence in Caco-2 cells untreated or treated with different concentrations of OE for 24h **(A)** and 48h **(B)**; and in Caco-2 cells untreated or treated with different concentrations of LE for 24h **(C)** and 48h **(D)**. Values are the mean ± SD of three independent experiments repeated at least in quintuplicate. Data were analyzed by 2-tailed Student's t test. $^*p < 0.05$; $^{**}p < 0.01$; $^{***}p < 0.001$; ns: non-significant.

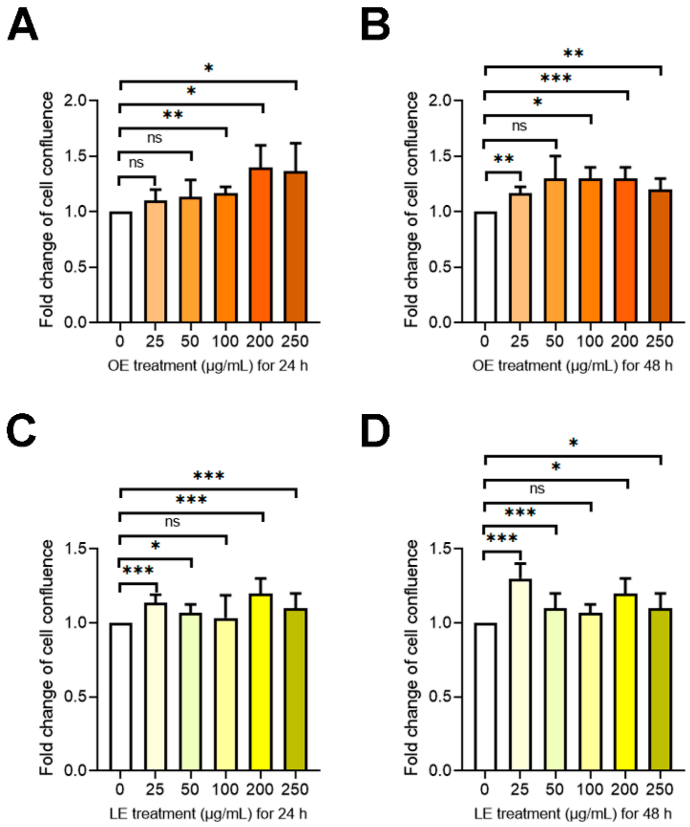

Supplement: Supplementary file 1 [file antioxidants-13-00869-s001.zip › antioxidants-3107147-supplementary.pdf]
